# RETRACTED ARTICLE: Study on the Impact of Inherent Ability on the High Quality of Life in the Elderly Based on Mediating Effect of Value Participation

**DOI:** 10.1007/s10803-023-05895-x

**Published:** 2023-02-11

**Authors:** Juan Luo, Xiaoxiao Chen, Yajun Duan, Yuliang Su

**Affiliations:** 1 School of Management, Shanghai University of Engineering Science, Shanghai, 201620 China; 2 School of Social Development and Public Policy, Fudan University, Shanghai, 200433 China

The Publisher has retracted this article in agreement with the Editor-in-Chief. The article was submitted to be part of a guest-edited issue. An investigation by the publisher found a number of articles, including this one, with a number of concerns, including but not limited to compromised editorial handling and peer review process, inappropriate or irrelevant references or not being in scope of the journal or guest-edited issue. Based on the investigation's findings the publisher, in consultation with the Editor-in-Chief therefore no longer has confidence in the results and conclusions of this article.

Author Juan Luo disagrees with this retraction. Authors Xiaoxiao Chen, Yajun Duan and Yuliang Su have not responded to correspondence regarding this retraction.

The online version of this article contains the full text of the retracted article as Supplementary Information.

### Electronic supplementary material

Below is the link to the electronic supplementary material.

Supplementary material 1 (PDF 695 kb)

