# Supplementary material for: RETRACTED ARTICLE: Study on the Impact of Inherent Ability on the High Quality of Life in the Elderly Based on Mediating Effect of Value Participation
Source: J Autism Dev Disord. 2023 Feb 11;54(4):1624. doi: 10.1007/s10803-023-05895-x (PMC9919750; doi:10.1007/s10803-023-05895-x)
Supplement: Supplementary file 1 — Supplementary material 1 (PDF 695 kb) [file 10803_2023_5895_MOESM1_ESM.pdf]

Journal of Autism and Developmental Disorders
https://doi.org/10.1007/s10803-023-05895-x

S.I. :IMPACT OF ASSISTIVE TECHNOLOGY IN SPECIAL EDUCATION

Check for updates

# Study on the Impact of Inherent Ability on the High Quality of Life in the Elderly Based on Mediating Effect of Value Participation

Juan Luo[1,2] · Xiaoxiao Chen[1] · Yajun Duan[1] · Yuliang Su[1]

Accepted: 4 January 2023
© The Author(s) 2023

**Abstract**
At present, China has entered the stage of rapid aging development, and the aging trend is becoming more and more serious. High-quality life has become an urgent concern of the society. Creating a high-quality life is the need to cope with the aging strategy and realize the high-quality development strategy. It is also the development trend of the elderly to pursue a high-quality life. This paper focuses on exploring the impact of inherent ability on the high quality of life using the psychological value participation of the elderly as the intermediary effect. Using logistic regression models to exam the relationship between the three independent variables, physical health and mental health and quality of life of the elderly, tests the robustness, and discusses the heterogeneity between gender and age to the conclusion of this paper. The results found that value participation and inherent ability have significant effects on high quality life, but on gender, age, household type and value participation, and the combination of individual social-economic factors and the environmental social support. This study enriches and expands the theoretical discourse of the inherent ability and high-quality life research of the Chinese elderly, and also provides the localization direction for the policy intervention of the high-quality life of the elderly based on the value, and is of great significance to build and improve the high-quality life in China's aging society.

**Keywords** High quality of life · Inherent ability · Value participation · The elderly · Physical health · Mental health and robustness

RETRACTED ARTICLE

## Introduction

At present, China has entered the stage of rapid aging development, and the aging trend is becoming more and more serious, which has become an urgent concern of the society. Growth is an improvement in order and a reduction in entropy, as well as the energy-driven synthesis of macromolecules from basic nutrients. Aging is a process of degradation, loss of order, and entropy development. Aging is related with increased positive emotional well-being, emotional stability, and emotional depth. The elderly may be less happy due to poor health; if the health aspect is removed, happiness may remain constant. On the other hand, one might argue that middle-aged people have higher earnings exactly due to the fact that they are older, which gives them more experience and, as a result, higher salaries compared to younger groups of people. According to the data released by the National Bureau of Statistics, there will be about 180 million people aged 65 and above in China in 2020, accounting for about 13% of the total population. By the time the 14th Five-Year Plan is completed in 2025, there will be more than 210 million people aged 65 and above, accounting for about 15% of the total population. In 2035 and 2050, the number of Chinese aged 65 or above will reach310 million and nearly 380 million, accounting for 22.3 percent and 27.9 percent of the total population, respectively. If the elderly population is defined as 60 years old and above, the number of elderly people in China will be even higher, approaching 500 million by 2050 (Table 1 and Fig. 1).

✉ Xiaoxiao Chen
chenx[illegible]@1[illegible]3.com

Juan Luo
emm[illegible]luojuan@163.com

Yajun Duan
june[illegible]526926@163.com

Yuliang Su
1901635227@qq.com

1 School of Management, Shanghai University of Engineering Science, Shanghai 201620, China

2 School of Social Development and Public Policy, Fudan University, Shanghai 200433, China

**Table 1** 2020–2050 forecast of population over 60 years old

| Year | Population over 60 years (%) |
|---|---|
| 2020 | 2.55 |
| 2030 | 3.71 |
| 2040 | 4.37 |
| 2050 | 4.84 |

Data are from Blue Book of Big Health Industry and China Business Industry Research Institute

The sixth plenary session of the 19th party stressed: to build a new pattern of development, promote high-quality development, comprehensively deepen reform and opening up, promote common prosperity… to accelerate the social construction focusing on improving people's livelihood, improve people's lives, cancel agricultural tax, constantly promote learning, labor, medical, old, living, promote social harmony and stability. Creating a high-quality life is the need to cope with the aging strategy and realize the high-quality development strategy. It is also the development trend of the elderly to pursue a high-quality life.

High-quality life is affected by many uncertain factors, such as the level of social and economic development, urbanization level, natural environment, resident income level, health level, etc. The degree of an elderly person's health is a factor that directly affects the quality of life of an elderly person and is referred to as the inherent ability. Inherent ability is the embodiment of a person's own ability, including both physical health and mental health. Social determinants of health for older persons, such as housing, food, financial resources, transportation, and social connections, are crucial for bettering overall wellbeing as well as improving health outcomes. Poor mental health and sometimes mental illness are often caused by diseases, injuries, and other physical issues. Some physical factors that directly modify brain chemistry and cause mental illness including birth trauma, brain damage, and unhealthy behaviors. It is the most important human capital for the elderly and a prerequisite for the social integration and participation.

High-quality life of the elderly not only requires “raising”, “medical”, “for”, “learning” and “music”.

But the pursuit of “health”, “love”, “use”, “into” and “enjoy” (Guangzong, 2019). With the improvement of material living standard, the basic life needs of the elderly to meet, the pursuit of the elderly has improved from the basic life needs to meet the spiritual needs, which mainly from mental, health, mental, psychological, social intangible abundance, and spiritual level needs can be achieved through social, personal and self-value participation (Glass et al., 2015). Therefore, value participation has also become an important influencing factor for the elderly.

This paper focuses on the value participation of the elderly as the intermediary effect, by building the inherent ability and value participation of the regression analysis model of the quality of life to explain how the inherent ability to achieve social value participation, spiritual value participation and labor value participation, to explore the influence of the inherent ability on the high quality of life in the elderly. Physical health, psychological state, independence, interactions with others, and one's surroundings are all necessary for a high-quality of life. Play the inherent ability of the elderly, improve the value of the elderly, can more systematically guide, lead, effectively promote China to further realize the aging society high quality life work goal, steady, steady, gradually promote to build quality life new heights required tasks, to build perfect high quality of old life theory framework and promote the implementation of the old quality of old life is of great significance.

The structure of the paper is discussed as follows:

Sect. “Literature Review” discusses about the literature review of the work followed by the introduction section; Sect. “Research assumptions and research methods” indicates the research assumptions and research methods; results and analysis of the research is discussed in Sect. “Research results and analysis”; finally, the conclusion of the research is discussed in Sect. Conclusion

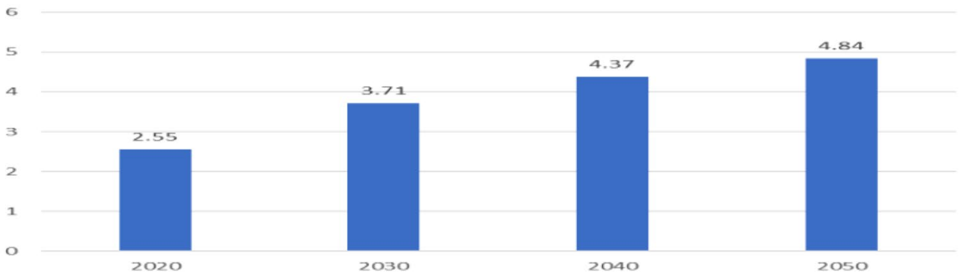


**Fig. 1** 2020–2050 forecast of population over 60 years old in China (100 million)

RETRACTED ARTICLE

## Literature Review

The academic circle has no unified definition of the concept of high-quality life and the evaluation dimension. From the perspective of evaluation characteristics, high quality life includes subjective and objective evaluation, subjective is mainly reflected in happiness, and objective is mainly reflected in the quality of life (Dongfang, 2021).The subjective research of the life happiness of the elderly is mainly concentrated in recent years. With the development of economy and society, the elderly's pursuit of life has become more and more rich, and the standards have been gradually improved. During the NPC (National People's Congress) and CPPCC (Chinese People's Political Consultative Conference) sessions in 2018, General Secretary Xi Jinping formally put forward the concept of "high-quality life", and compared it with "high-quality development". Scholars' research on the life of the elderly began to focus on the research of high-quality life and compiled a high-quality life evaluation index system in Shanghai, dividing the high-quality life into three target levels: "sense of gain", "happiness" and "security". Scholars study how to improve the life happiness of the elderly from different perspectives. Among them, the factors affecting the living happiness of the elderly are their mobility, education level, economic level, source of life, timely arrival at the hospital, routine physical examination, and community support (Yue et al., 2021).Among them, family care has a significant impact on the li[illegible]appiness of the elderly, which can reduce the depression de[illegible]e of the elderly and increase the social activit[illegible] ch[illegible]nels (Feng & Fu, 2017).For elderly people aged 6[illegible] or olde[illegible] studies show that family relationships, leisure activities, and living and housing conditions play a daily [illegible]le in improving the quality of life and life well-being (Wong, [illegible]019).

Objectively on the quality of [illegible] the elderly, the influencing factors of the elderly from different dimensions. Internationally, in 1958, the [illegible].K.Ca[illegible]brith first mentioned the quality of life in his b[illegible] [illegible]ch Society". In the 1980s, China began to [illegible]eeply s[illegible]y the evaluation system and methods to impro[illegible] residents' quality of life. Most studies at home an[illegible] abroad [illegible]inly study the factors affecting the quality of life of the elderly, including demographic factors: gender, ag[illegible]education, marital status, social insurance, living ar[illegible]ngeme[illegible], income, social support status, health status, e[illegible] (Y[illegible] 2011; Borg & Hallberg, 2006). How to evaluate t[illegible] quality of life level, the World Health Organization published the Quality of Life Scale (WHO QOL) in 1991, involving a comprehensive index evaluation system of 24 aspects in six areas. In the early 2000 century, the World Health Organization once again proposed the concept of "active aging", focusing once again on the quality of life of the elderly. Quality of life is a multidimensional concept, including physical and mental health, cognitive and self-care ability, social conditions, economic conditions, and life satisfaction (Chen& Wu, 2021; Zimmermann, 1991), and economic development, cultural and regional differences.

Inner ability is an important influencing factor when studying quality of life. The inherent ability is [illegible] combination of all physical functions and mental power [illegible]t individuals can use at all times, such as physical health [illegible]nd mental health that can enable individual act[illegible] (Global Report on Aging and Health, 2016; Decade of Hea[illegible]y Aging Action 2020–2030, 2020).In the stu[illegible]y on the impact of inherent ability on the high qua[illegible]y o[illegible]ife of the elderly, foreign scholars call it functi[illegible]n (S[illegible]za Macedo, 2021; Lin et al., 2022), and family f[illegible]ction is [illegible]ositively correlated with the quality of life of [illegible]e el[illegible]ly (Souza Macedo, 2021). Physical function has [illegible] associated with depression and ability to daily tools [illegible] sig[illegible]ficantly associated with depression, with a higher risk [illegible] death despite better health and functional perfor[illegible]ance (Li[illegible] et al., 2022). Significant effects of chronic diseases o[illegible] [illegible]ity of life in older adults, (Campolina, 2011). When domestic scholars study their inherent abilities, they divide them into mental health and physical health, and con[illegible]ct a comprehensive analysis (Li Chunyu et al., 2021).The first criterion to measure the health of the elderly is function, [illegible]ather than blindly pursuing the absence of disease (Shan, 2021), and the mental health of the elderly is related to subjective happiness (Linfeng et al., 2020).Studies have shown that the overall health level of the elderly is poor, and more attention should be paid to their ability to take care of themselves in their daily life (Yan Wei, 2021).Self-care ability is the most common problem affecting the health and quality of life of the elderly (Xiaolan & Ji et al., 2018).Improving the physiological health level of the elderly can effectively promote the life happiness of the elderly (Jiang et al, 2018). Compared with healthy people, the mental health level of the elderly with serious diseases is relatively low, and with the increasing severity of the disease, the mental health level of the patients also decreases, so the physical health status has a positive impact on the mental health of the elderly (Jiaming et al., 2016).Academic circles about the elderly physical health, self-care ability and mental health research shows that physical health and mental health on the subjective happiness of the elderly, the elderly physical health mental health, it has the inherent conditions to make individual action, the life happiness, the quality of life.

In addition to the inherent ability of older people, value participation is another class of important influencing factors worth studying. Value participation is generally a

RETRACTED ARTICLE

psychological term, namely value intervention or value guidance, which refers to the process in which counselors influence and change the visitor's values to varying degrees (Wu Huang et al., 2021). The definition of value participation described in this article is closer to social participation, so the connotation of value participation will be explained through the social participation of the elderly.

Social participation is a behavior, while value participation is a psychological and spiritual satisfaction, and value participation is achieved through social participation. Value participation of the elderly generally refers to all the beneficial activities that the elderly participates in that contribute to the society or others, but also can meet the income source, spiritual support and self-value realization of the elderly. There are various kinds of forms of value participation for the elderly, including employment, political participation, housework, intergenerational care, volunteer activities, and education and training (Wang et al., 2015). The categories of participation forms also show diversity according to the needs of each research. Some scholars divide them into four categories: work, leisure, social and housework based on the form of employment, participation in organizations for the elderly, volunteer activities and housework time, while some scholars divide them into market economic activities and non-market activities with economic value based on the absence of remuneration. In general, related studies often reveal various aspects of the form of elderly value participation, without summarizing and unifying their general model (Zhang & Zhao, 2015).In recent years, scholars at home and abroad have begun to study the relationship between some and two types of value participation, such as volunteer service and economic participation, intergenerational care and volunteer service, intergenerational care and economic participation (Bulanda & Jendrek, 2016; [illegible] et al., 2018).A small number of foreign scholars gradually began to pay attention to the synchronicity of the elderly participation in various fields and the resulting value participation model formed. By analyzing the frequency of elderly participation in 20 activities, elderly value participation patterns were divided into high participation, active leisure, passive leisure, and low participation. Domestic research for the elderly value participation model is lagging behind, through the elderly to participate in economic activities, social activities, political activities and family activities, through the potential category model of the domestic elderly value participation models into three types, namely high participation, low participation and family care (Xie, Wang, 2019).

Furthermore, many scholars have found that positive value participation in seniors is beneficial to their subjective well-being or mental health, specifically, sustained value participation may be more closely associated with fewer depressive symptoms (Shiba et al., 2021) and alleviates the deterioration of self-rated health and decreased life satisfaction (Eunjin et al., 2021).Promoting value participation of various types and appropriate frequencies may reduce physical weakness in the elderly (Xie & Wang, 2019).Moreover, the value participation in the elderly can also have a significant impact on self-efficacy (Park et al., 2022), which can help enhance happiness and then help the elderly people realize the value of life (Wang et al., 2015).Especially under the global impact of COVID-19, the elderly population's value participation is more limited ([illegible] 202[illegible]).Elderly can be brought to a higher quality of life by encouraging them to participate more in social activities (Palmes et al., 2021). Returning community activities and promoting participation of the elderly and taking full consideration of infection prevention is an important means of maintaining mental health in the elderly (Noguchi et al., 2022).

Based on previous studies of domestic and foreign scholars, intrinsic ability and value participation are significantly associated with the health of the elderly, however, the existing research mainly focuses on its impact on the individual health of the elderly, with little attention on the impact of individual development and relations with the elderly. At the same time, the division of the realization path of high-quality life of the elderly is mostly based on various factors such as social, economic, leisure, culture, family and so on. The generalization of it into the lack of inherent ability and value participation and making specific analytical research also provides room for in-depth discussion in this paper.

## Research Assumptions and Research Methods

### Research on Assumptions and Theoretical Models

Creating a high-quality life is the need to cope with the aging strategy and realize the high-quality development strategy. Through sorting the academic research, the quality of life and life happiness are selected as the measurement indicators of the high-quality life of the elderly. Quality of life can measure people's living standard, as an objective index. Life happiness can reflect people's satisfaction with the life status quo, which is a subjective index. Subjective indicators and objective indicators are combined to more systematically evaluate personal life. Subjective indicators are information that contains some type of a subjective component, such as a personal perspective or a personal assessment. Subjective indicators are sometimes characterized as information that includes some kind of a subjective component. This concept is thus exclusively concerned with the subjective evaluation

RETRACTED ARTICLE

of a criteria of any sort. An objective indication is often considered to have a higher degree of reliability than a subjective evaluation. A marker or other measure of an entity, state, emotion, or action that is devoid of any subjective bias; to put it another way, it is not an opinion or rating but instead an independent measure. This paper discusses the influencing factors of the inherent ability index, and considers the intermediary role of the value participation index in it.

Inherent ability refers to the individual's physical function, including mental power, physical strength and other ability to enable the individual to maintain daily behavior. Inherent ability of the elderly has a crucial impact on their life well-being (Marguerite, 1992). As we get older, we go through an increasing number of significant life changes, such as changing careers or retiring, children leaving home, the loss of loved ones, physical and health challenges, and even a loss of independence. Some of these changes can occur simultaneously, while others occur one after the other. How we respond to change and how we mature as a result of it are typically the determining factors in healthy aging. A healthy body can promote the healthy life span of the elderly, and improve the quality of life and well-being of the elderly. Inherent ability depends on a number of factors, including physiological and psychological changes as a basis, health-related behaviors, and illness or not. Based on the above research basis, this paper proposes the research hypothesis 1:

**H1** The Inherent Ability of the Elderly is Significantly and Positively Correlated with Their Quality of Life and Life Happiness

The value participation of the elderly refers to the health-related factors that the elderly can live and act according to their own ideas and preferences, including their social participation, family participation, and education for the elderly, which can give full play to their own value, and can meet the spiritual needs of the elderly. People who often participate socially are more able to realize their own value and have a higher quality of life (Skla[illegible] al., 2021). Most elderly people believe that a rich social life can slow down their loneliness and improve their happiness in life (Noel et al., 2017). Based on the above research basis, the research hypothesis 2:

**H2** The Value Participation of the Elderly is Significantly Positively Related with Their Quality of Life and Happiness

Individual characteristic factors of the elderly will affect the value participation of the elderly, with individual characteristic factors including their physical quality, social status, etc. (Brusilovskiy et al., 2020), Older people in better health, willing to socialize and help others are more inclined to value participate(Yuehua et al., 2017). Therefore, the inherent ability of the elderly will have an impact on their life happiness and quality of life by affecting the value participation process of the elderly. Based on the above research basis, this paper proposes the hypothesis 3:

**H3** The Value Participation of the Elderly Plays an Intermediary Role in the Influence of the inherent Ability on Their Quality of Life and Life Happiness, that is, the Inherent Ability of the Elderly Affects Their Quality and Life Happiness Through the Quality of Life and Value Participation

To validate the results, the outcome robustness test was performed. Robust testing focuses on enhancing dependability and identifying edge cases by entering data that simulates extreme environmental circumstances to assess whether or not the system is robust enough to perform. The testing of robustness is more targeted than the benchmarking of dependability. Family relationships, friends, and neighbor relationships had a significant influence on subjective well-being ([illegible]ury s, et al., , 2013), Life satisfaction is an important means of assessing the quality of life in the elderly population(Helliwell & Putnam, 2004), Therefore, in this paper, the harmony of family membership instead of happiness in life, and quality of life instead of life satisfaction to analyze the robustness of the results, thus proposing the hypothesis 4:

**H4** The Harmony of Family Membership is Used Instead of Life Happiness, and Quality of Life Satisfaction as a Measurement Variable of High Quality of Life in the Elderly, and the Results Are Still Significant

With the growth of age, the physical function of the elderly gradually appear signs of decline, excessive participation in social labor will increase the physical burden of the elderly, but also increase the probability of the elderly to fall, resulting in greater physical damage(Brusilovskiy et al., 2016; Zhai, 2016). In order to improve their quality of life, the elderly still need to often participate in social labor, which leads to the decline of their physical function; and the elderly who appropriately or do not participate in social labor, the higher the quality of life, the better their physical condition. So, this paper proposes the hypothesis that the 5:

**H5** Social Labor Participation Will Affect the Correlation Between Physical Health and Quality of Life of The Elderly

The quality of life and physical function were both improved in physically active people. A better quality of life for the elderly might be achieved by the early diagnosis of a decrease in their physical function as well as an increase

RETRACTED ARTICLE

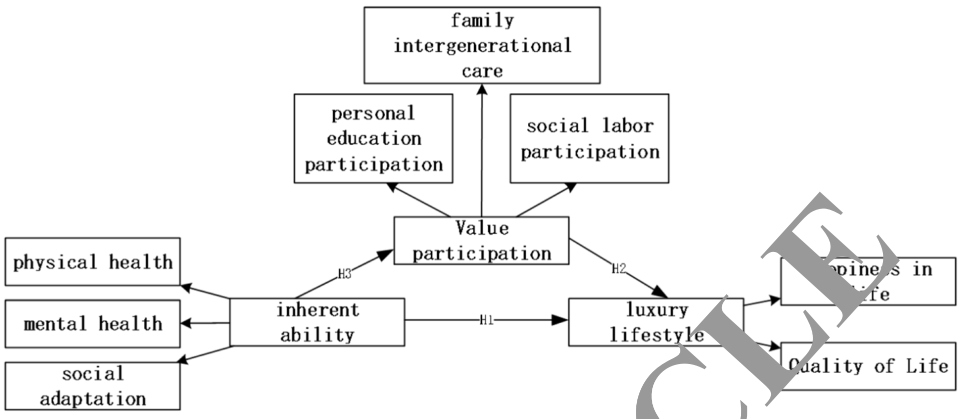


**Fig. 2** The theoretical framework

**Table 2** Distribution of the survey areas

| Research area | Scale | Research area | Scale |
|---|---|---|---|
| Huangpu district | 6.5% | Baoshan District | 7.9% |
| Xuhui District | 6.8% | Jiading district | 4.6% |
| Changning District | 3.9% | Pudong New Area | 17.4% |
| Jing'an District | 7.2% | Jinshan district | 4.0% |
| Putuo district | 6.3% | Songjiang district | 3.8% |
| Hongkou district | 5.2% | Qingpu area | 3.2% |
| Yangpu district | 8.1% | Fengxian district | 3.7% |
| Minhang District | 5.4% | Chongming district | 4.6[illegible] |

in their level of physical activity. Therefore, the th[illegible] research framework in Fig. 2:

## Research methods and data sources

In this study, the cognition and nee[illegible] of hig[illegible]-quality life were analyzed by sampling the elder[illegible] Shanghai. This data is obtained from 64 peop[illegible]m 32 research groups through a two-month interview q[illegible]stion-and-answer survey of the elderly in 16 districts [illegible]f Shanghai. According to 2020, a total of 2700 questi[illegible] were issued from 0.5 ‰ in Shanghai over 60 years o[illegible] and 2572 valid questionnaires were actually r[illegible]ed, with an efficiency as high as 95.2%. The labor [illegible]st of ea[illegible] questionnaire was 10 yuan, and a small gif[illegible] was prepared for each respondent.

The sp[illegible]c cir[illegible]umstances are shown in Table 2:

### D[illegible]en[illegible] Variable

A depe[illegible]dent variable is a factor whose value is determined by the interaction of many other variables. For instance, a test score may be considered a dependent variable since it might alter based on a number of different aspects, such as how much time users spent studying for the test. The dependent variables in this study were the well-being and quality of life in older adults. Dependent variable is the answers to the two q[illegible]: "How do you evaluate your quality of life" and "[illegible]o you th[illegible]k you live a happy life now". The answers are [illegible]et to [illegible]ptions For life happiness, they are divided into "[illegible]piness = [illegible]", "unhappy = 0"; for quality of life, "high [illegible]alit[illegible]" and "low quality = 0".

### Argu[illegible]nt

The inde[illegible]endent variable in this study was the inherent ability of older adults. According to the three relevant ques[illegible]ns in the questionnaire, three measurement variables of th[illegible] inherent ability of the elderly were selected, namely "[illegible]iving and physical health status of the elderly", "physical health status of the elderly" and "recognition of the obtained mental health status". There is a hierarchical relationship between the anwers to the three questions (5 = fully able to take care of themselves, very healthy, very agree, 1 = completely unable to take care of themselves, very unhealthy, and completely disagree).

### Metavariable

Value participation of the elderly involves social, family, individual dimensions, according to the relevant three questions in the questionnaire, select three elderly value participation measurement variables, respectively is "elderly social labor participation (1 = participation, 2 = not participate)", "elderly family intergenerational care (1 = need to care for grandchildren, 2 = no need to take care of grandchildren)", "the elderly in elderly education participation (1 = participate in elderly education, 2 = do not participate in elderly education)". In terms of personal effects, social labor participation helps elderly individuals maintain their physical and mental health and fights against dementia.

### Controlled Variable

Different levels of economic development in different places, different levels of public services for the elderly lead to



RETRACTED ARTICLE

RETRACTED ARTICLE

**Table 3** Description and descriptive statistics of the various variables

| Variable's attribute | Variable name | Type of variable | Sample number | Description of the variables and the descriptive statistics |
|---|---|---|---|---|
| dependent variable | Happiness in life | Classification | 2572 | Satisfied = 1, Unsatisfied = 0 |
| | quality of life | Classification | 2572 | High quality = 1, low quality = 0 |
| Argument (innate capacity) | physical condition | Classification | 2572 | Maximum = 5, minimum = 1 |
| | social adjustment | Classification | 2572 | Maximum = 5, minimum = 1 |
| | Mental health status | Classification | 2572 | Maximum = 5, minimum = 1 |
| Metavariable (Value participation) | Social labor participation | Classification | 2572 | Participation = 1; No participation = 0 |
| | Family care across generations | Classification | 2572 | No care required = 1; [illegible] care required = 0 |
| | Personal education participation | Classification | 2572 | Participation = 1; No participation = 0 |
| Controlled variable (personal feature) | domicile | Classification | 2572 | Urban area = 1; Suburban = 0 |
| | Age | Classification | 2572 | 60–70 years = [illegible]; 71–80 years = 2; 81–90 years = [illegible]; [illegible] years and older = 4 |
| | Sex | Classification | 2572 | Female = 1; [illegible]ale = 0 |
| | Cultural standing | Classification | 2572 | Primary school and below = 1; junior high school = 2; Technical secondary school or high school = 3; junior college or above = 4 |

different resources, affecting the happiness and quality of life, different age background, the cognitive influence of gender on people always exists, different sexes have different views, the way and vision of the culture, leading to different views of the same event. Based on this, in this study, four variables: the residence, household registration, sex and cultural level of the interviewed elderly people were set as control variables.

Detailed description of the above variables and some descriptive statistical analysis are shown in Table [illegible]

## Statistical Model

This paper examines the relationship between the three independent variables, physical health and mental health and quality of life of the elderly, tests the robustness, and discusses the heterogeneity between gender and age to the conclusion of this paper.

## Research Results and Analysis

### Analysis of the Regression Results

A logistic regression analysis model of the inherent ability and value participation of the independent variable elderly people on their quality of life was constructed, which showed that gender, cultural level, and age had significant effects on the quality of life of the older people. A statistical analysis approach called logistic regression uses previous observations from a data set to predict a binary result, such as yes or no. By investigating the relationship between one or more previously present independent variables, a logistic regression model estimates a dependent data variable. According to model 1, in the control variables, the gender, cultural level, and age of the elderly all have a significant impact on the quality of life of the elderly. Secondly, the participation of social labor has a significant impact on the quality of life of the elderly. It is known from model 2 that the social labor participation in the independent variable value participation has a significant impact on the quality of life of the elderly. However, family intergenerational care and personal education participation did not show significance for the quality of life of the elderly. Third, the inherent ability of the elderly significantly affects the quality of life of the elderly. It can be seen from model three that the three variables in the inherent ability of independent variables: social adaptation, physical health status and mental health status all have a significant impact on the quality of life of the elderly. Finally, the significance of social labor participation on the quality of life in the elderly disappeared when the inherent competence, value participation were jointly included in the model. It can be seen from model 4, when the inherent ability and value participation are included in the model, the significance of social labor participation on the quality of life of the elderly disappears, but the three variables in the inherent ability are still highly significant on the quality of life of the elderly Table 4.

A logistic regression analysis model of the inherent ability and value participation of independent variable elderly people on their well-being in life was constructed, which showed that the residence, gender and cultural level had a significant impact on their well-being. As seen from model

**Table 4** Logistic regression model of quality of life (objective)

| | Model 1 | Model 2 | Model 3 | Model 4 |
|---|---|---|---|---|
| Argument | | | | |
| Innate capacity | | | | |
| Social adjustment | | | 0.314*** | 0.306*** |
| Physical condition | | | 0.226*** | 0.221*** |
| Mental health status | | | 0.561*** | 0.562*** |
| Value participation | | | | |
| Social labor participation | | 0.295** | | [illegible].149 |
| Family care across generations | | 0.057 | | 0.028 |
| Personal education participation | | 0.067 | | – 0.014 |
| Controlled variable | | | | |
| Domicile | – 0.110 | – 0.105 | – 0.285** | – 0.281** |
| Sex | 0.151* | 0.146* | 0.094 | 0.093 |
| Cultural standing | 0.412*** | 0.403[illegible] | 0.332*** | 0.331*** |
| Age | 0.256*** | – 1.364*** | 0.387*** | – 5.236*** |
| Negolco R square | 0.065 | [illegible]70 | 0.165 | 0.166 |
| -2 log-likelihood | 3232.197 | 3223.36[illegible] | 3232.435 | 3030.640 |

*p < 0.1, **p < 0.05, ***p < 0.01, and the coefficient is the standard error estimated in parentheses

**Table 5** Logistic regression model of life happiness (subjective)

| | Model 5 | Model 6 | Model 7 | Model 8 |
|---|---|---|---|---|
| Argument | | | | |
| Innate capacity | | | | |
| Social adjustment | | | 0.352*** | 0.349*** |
| Physical condition | | | 0.360*** | 0.358*** |
| Mental health status | | | 1.040*** | 1.052*** |
| Value participation | | | | |
| Social labor participation | | 0.123 | | – 0.005 |
| Family care across generations | | 0.280** | | 0.122** |
| Personal education participation | | – 0.287* | | – 0.216* |
| Controlled variable | | | | |
| Domicile | – 0.582*** | – 0.580*** | – 0.948** | – 0.964*** |
| Sex | 0.371** | 0.359** | 0.359* | 0.360* |
| Cultural standing | 0.349*** | 0.349*** | 0.202** | 0.219*** |
| Age | – 0.008 | 0.018 | 0.160 | 0.177 |
| Negolco R square | 0.051 | 0.053 | 0.229 | 0.230 |
| -2 log-likelihood | 1633.510 | 1631.393 | 1399.900 | 1397.703 |

*p < 0.1, **p < 0.05, ***p < 0.01, and the coefficient is the standard error estimated in parentheses

RETRACTED ARTICLE

5, the residence, gender and cultural level of the elderly in the control variables all had a significant impact on the life well-being of the elderly. Secondly, family intergenerational care and participation in personal education have a significant impact on the well-being of the elderly. We can see that the family care and personal education participation have a positive impact on the life happiness of the elderly. Third, the inherent ability of the elderly significantly affects the life happiness of the elderly. It can be seen from model 7, the three variables of the independent variable: social adaptation, physical health status and mental health status all have a significant impact on the life happiness of the elderly. Finally, inherent ability, intergenerational care, and participation in education for the elderly have a significant impact on the life well-being of the elderly. According to model eight, the three variables in the inherent ability of the elderly, as well as the family intergenerational care and personal education participation in value participation, all had a significant impact on the life happiness of the elderly, while the elderly social labor participation in value participation did not show a significant correlation Table 5.

**Table 6** Endogenous issues

| Variable name | Quality of life | Happiness in life | Social labor participation | Family care across generations | Personal education participation |
|---|---|---|---|---|---|
| Innate capacity | | | | | |
| Social adjustment | 0.188*** | 0.118*** | 0.143*** | 0.210*** | 0.157*** |
| physical condition | 0.288*** | 0.165*** | 0.169*** | 0.089* | 0.264*** |
| Mental health status | 0.292*** | 0.011 | 0.043 | – 0.210*** | 0.332*** |
| Controlled variable | YES | YES | YES | YES | YES |
| F-value | 18.988 | 17.302 | 17.243 | 17.052 | [illegible] |

RETRACTED ARTICLE

### Inherent Ability Can Significantly Improve the Quality of Life of the Elderly

According to Tables 4 and 5, inherent ability has a significant positive impact on the quality of life and life wellbeing of older people. Thus, Hypothesis 1 is verified. The physical health, mental health and social adaptation of the elderly have a steady and significant impact on their daily life and subjective well-being. Health status indicators such as physical health and mental health have a positive effect on the happiness of the elderly, and poor health status has a negative impact on the happiness of the elderly. In addition, social adaptation not only has a direct beneficial effect on the physical and mental health of the elderly, but also makes the elderly enter a new life cycle after retirement. Due to the role change, the elderly is easy to have a sense of “[illegible]ment”. elderly who successfully adapt to old life can [illegible] themselves alive and strive not to withdraw [illegible] social life. Therefore, if the elderly can maintain as long activities as possible, they can adjust and adapt to later life, and feel satisfied and happy with later life. Therefore, when promoting the improvement of the living quality [illegible] of the elderly, special attention should be paid [illegible] improvement of their inherent ability.

### Value Participation Has a Greater Impact on the Subjective Happiness of the Elderly

From Tables 4 and 5, value participation was more significant for [illegible] wellbeing of older people, and hypothesis 2 was [illegible]fied. Therefore, value participation can affect the life [illegible] the elderly more than the quality of life of the elderly. The main reason for value participation result is that most of the elderly people will feel idle and feel lonely after retirement. Family care can make grandparents more self-efficacy, and even lead them to a healthier and positive lifestyle, such as quitting smoking. At the same time, the interaction between grandparents and grandchildren can enrich their later life, play the role of teaching the next generation, effectively alleviate the negative psychological impact, and improve their subjective happiness. In addition, when the elderly participated in social [illegible] and personal education, they can reduce their loneliness, increase the communication with the society, give full play to their social value, and enhance their subjective happiness.

### The Endogenous Testing

In the above analyses, inherent abilities may have endogenous problems. For example, older adults may increase social, family and personal value engagement while [illegible] quality of life and life well-being, thus also affecting changes in inherent abilities, which will lead to bias in estimated outcomes. To address this issue, this study used pre-retirement occupation as an instrumental variable for inherent competence, estimated using the two-phase least squares (2SLS) model, with regression results shown in the Table 6. The study of structural equations often makes use of a statistical method known as the Two-Stage Least Squares, or 2SLS Regression Analysis. This method is an extension of the ordinary least squares approach. It is used in situations in which the error terms of the dependent variable are correlated with those of the independent variables. It is also helpful when the model has feedback loops. The F values estimated at one stage were all greater than 10, with no problem of weak tool variables. After correcting for endogenous bias, social adaptation, physical health, and mental health status on quality of life, life wellbeing, and value participation are consistent with those mentioned above, supporting the hypothesis proposed in this study Table 6.

### Test of Robustness

To test the robustness of the above results, the criteria for assessment of happiness and quality of life. From the data itself, the higher the happiness of older people with harmonious family relations and the higher satisfaction with quality of life. Therefore, if the previous conclusion is true, the regression results after replacement with new variables should be basically consistent with the previous results.

RETRACTED ARTICLE

**Table 7** Test of robustness

| | Happiness in life | Quality of life |
|---|---|---|
| Innate capacity | | |
| Social adjustment | 0.439*** | 0.295*** |
| Physical condition | 0.290*** | 0.357*** |
| Mental health status | 0.911*** | 0.379*** |
| Value participation | | |
| Social labor participation | 0.398 | 0.030 |
| Family care across generations | 0.280** | 0.367 |
| Personal education participation | − 0.287* | 0.130 |
| Controlled variable | YES | YES |
| Constant | − 4.686*** | − 3.877*** |
| -2 log-likelihood | 1503.922 | 2624.143 |
| Chi-square | 262.427*** | 195.929*** |

*p < 0.1, **p < 0.05, ***p < 0.01, and the coefficient is the standard error estimated in parentheses

In this questionnaire, the family member harmony was chosen to replace life happiness. 0 and 1 according to the answer setting represent member disharmony and member harmony; choose life satisfaction to replace quality of life, 0 and 1 points according to the answer setting represent low life satisfaction and high life satisfaction. As a new measure of high-quality life in aging society, the regression steps were repeated and the results are consistent with the original. Details are given in Table 7:

## Heterogeneity Analysis

According to the characteristics of the respondents, gender, age, whether to participate in social labor, participate in the elderly education of heterogeneous testing, 60–70 as young, 71–80 as aged, 81–[illegible] as elderly, over 90 as super elderly. The regression results are shown in the table below Table 8 and 9.

**Table 8** Heterogeneity analysis of the—subpopulations

| | Sex | | Age | | | |
|---|---|---|---|---|---|---|
| | the male sex | Femininity | Low age | Middle age | Advanced age | Super advanced age |
| Happiness in life | | | | | | |
| Innate capacity | | | | | | |
| Social adjustment | 0.322** | 0.364 | 2.440 | 0.500*** | 2.113 | 0.298 |
| Physical condition | 0.270** | 0.415*** | 0.356*** | 0.551*** | 1.125*** | 0.400 |
| Mental health status | 1.155*** | [illegible] | 1.010*** | 1.069*** | 1.943*** | 2.801* |
| Value participation | | | | | | |
| Social labor participation | 0.830 | 2.075 | 0.135 | 0.019 | 0.010 | 0.212 |
| Family care across generations | 0.345 | 0.203 | 0.566 | 1.880 | 0.159 | 0.088 |
| Personal education participation | 0.207 | 1.666 | 0.677 | 0.033 | -1.678** | 0.132 |
| Controlled variable | YES | YES | YES | YES | YES | YES |
| Constant | 1.960*** | 2.201*** | 2.119*** | 2.114*** | 1.817*** | 1.335*** |
| -2 log-likelihood | 705.964 | 689.855 | 812.967 | 454.453 | 102.354 | 15.362 |
| Chi-square | 147.744*** | 149.701*** | 121.137*** | 129.237*** | 48.147*** | 9.201*** |
| Quality of life | | | | | | |
| Innate capacity | | | | | | |
| Social adjustment | 0.327*** | 0.307** | 0.389*** | 0.206* | 0.436* | 1.655 |
| Physical condition | 0.222** | 0.246** | 0.214** | 0.275*** | 0.641 | 0.522 |
| Mental health status | 0.616*** | 0.532*** | 0.460*** | 0.555*** | 1.072*** | 15.137*** |
| Value participation | | | | | | |
| Social labor participation | 0.722 | 0.991 | 0.011 | 2.536 | 0.688 | 1.412 |
| Family care across generations | − 0.329** | 0.358*** | 0.978 | 0.236 | 0.255 | 1.008 |
| Personal education participation | 1.063 | 1.086 | 0.274 | 0.565 | 0.465 | 0.898 |
| Controlled variable | YES | YES | YES | YES | YES | YES |
| Constant | 0.193*** | 0.195*** | 0.112** | 0.216*** | 0.621*** | 0.887** |
| -2 log-likelihood | 1420.695 | 1595.945 | 1720.907 | 1079.817 | 199.135 | 0 |
| Chi-square | 151.842*** | 185.631*** | 175.376*** | 94.148*** | 41.591*** | 28.975*** |

*p < 0.1, **p < 0.05, ***p < 0.01, and the coefficient is the standard error estimated in parentheses

### Family Value Participation Can Significantly Improve the Life Happiness of the Elderly. Family Value Participation is More Important than Social Value Participation and Personal Value Participation. Women Think Their Quality of Life is Higher When Their Family Values Participate

As can be seen from Table 9, the elderly people participating in local rural areas, local urban areas, rural areas and rural towns are positively correlated with their living happiness. At the same time, Table 8 shows that compared with social value participation and personal value participation, family value participation is more significantly associated with the life happiness and quality of life of the elderly. The main reason for this result is that although social labor participation and education participation for the elderly can improve the income and reduce their loneliness to a certain extent, thus improving their subjective happiness to a certain extent. However, the pension income level of the elderly in some places (such as Shanghai) is relatively high, and the economic income brought by social labor participation can not make them have more happiness. It is more like the education participation of the elderly, which more alleviates the psychological loneliness of the elderly. When taking intergenerational family care, the elderly not only directly improve their subjective happiness, but also indirectly affect their subjective happiness through economic support, emotional comfort and social communication with their children. In conclusion, although participation in family intergenerational care will reduce the social labor participation and personal education participation of the elderly, the elderly will emotionally receive more support from their children. Therefore, for the elderly in some places, family value participation is more important than social value participation and personal value participation. In addition, for female elderly, sensibility is generally more than rational, so female elderly people are more likely to obtain emotional satisfaction, which explains [illegible] women will think that they have higher quality of life when participating in family value (]Zhiwei Lian et al., 2021; Fuqun Xiao & Li, 2021; He et al., 2020; Li & Liu, 2015; Lima et al., 2020 Jul 29; Liu & Peng, 2019; Townley et al., 2017, 2018, 2019).

### When Promoting the Social Value Participation of the Elderly, the Gender Differences and Physical Health Differences of the Elderly Should Also Be Fully Taken into Account

As shown from Table 9, women and elderly people who did not participate in social labor were significantly associated with their life happiness and showed a positive correlation. That is, for female elderly people, they think it is happier not to participate in social labor life. The emergence of this result may be related to the social value participation rate, who have significantly lower social participation than older men. This is closely related to the long-term care of female elderly families. Therefore, female older people are also more willing to return to their families rather than participate in social labor in old age. In addition, compared with the elderly who participated in social labor, the physical health status of the elderly who did not participate in social labor was significantly associated with their quality of life, and showed a positive correlation. For the elderly who participate in social labor, their quality of life is negatively related to their physical health status. In other words, when promoting the social value participation of the elderly, the physical health differences should also be fully considered in the elderly. This point is also consistent with the reality, health is the basic factor to improve the quality of life, the elderly can have a healthy body is one of the basic conditions for social value participation. At the same time, through long-term exercise, the elderly can not only improve their physical health, but also expand their personal social network, promote mental health, and then conduct wider social value participation, and then improve the quality of life and happiness of the elderly. This also verifies the hypothesis 5 presented in this paper (Choy-Brown et al., 2020).

### The Higher the Cultural Level, the Higher the Life Happiness

By Table 8, compared with the elderly to participate in social labor, not involved in social labor of their cultural level and life happiness has no significant correlation, but participate in the social labor of the elderly cultural level and their life happiness, that is to say, the elderly to participate in social labor, the higher the cultural level, the higher the life happiness. The reason for this result is that literacy is positively associated with subjective well-being in older adults. Generally speaking, compared with those who have not been to school, the elderly with a certain learning experience and a certain culture has a higher level of subjective happiness. With the improvement of academic qualifications, the subjective happiness level of the elderly individuals will be further improved. So, for the elderly in some places (such as Shanghai), their cultural level is generally relatively high, and the higher their cultural level, it is easier to realize their own value when participating in social labor, thus improving their life happiness.

### There are Obvious Differences in the Quality of Life of the Elderly in Urban and Rural Areas

Similarly, older people who did not participate in individual education, older people living in the suburbs showed a negative correlation with their quality of life. The point is also in line with the reality. When Jianxin Li and other

RETRACTED ARTICLE

**Table 9** Heterogeneity analysis of the—subpopulations

| | Whether to participate in social labor | | Whether to participate in intergenerational care | | Whether to participate in the elderly education | |
|---|---|---|---|---|---|---|
| | Yes | Deny | Yes | Deny | Yes | Deny |
| Happiness in life | | | | | | |
| Innate capacity | | | | | | |
| Social adjustment | 0.621* | 0.321** | 0.344* | 0.356* | 0.267[illegible] | 0.370** |
| Physical condition | 0.472* | 0.340*** | 0.476** | 0.260* | 0.402* | 0.372** |
| Mental health status | 1.013*** | 1.079*** | 1.111*** | 0.994*** | 0.983*** | 1.075*** |
| Controlled variable | | | | | | |
| Current residence (urban area) | − 1.236** | − 0.853*** | − 1.305*** | − 0.668*** | − 1.6[illegible]** | − 0.811*** |
| Household type (local rural) | 0.238 | 0.100 | 0.330* | − 0.131 | 0.152 | 0.095 |
| Gender (male) | 0.097 | 0.418** | 0.225 | 0.[illegible]92* | 0.202 | 0.396* |
| Cultural level (primary school and below) | 0.600*** | 0.126 | 0.207* | 0.2[illegible] | 0.254 | 0.196* |
| Age | 0.463 | 0.116 | 0.199 | 0.146 | 0.156 | 0.175 |
| Constant | − 6.349*** | − 3.847*** | − 4.329*** | [illegible].684*** | − 2.771* | − 4.376*** |
| -2 log-likelihood | 217.972 | 1168.456 | 619.424 | 768.[illegible]98 | 273.894 | 1118.711 |
| Chi-square | 80.172*** | 227.759*** | 155.[illegible]3*** | 152.320*** | 53.891*** | 249.028*** |
| Quality of life | | | | | | |
| Innate capacity | | | | | | |
| Social adjustment | 0.481** | 0.274*** | 0.[illegible]** | 0.278** | − 0.088 | 0.412*** |
| Physical condition | − 0.034 | 0.270*** | 0.189* | 0.272** | 0.404** | 0.199** |
| Mental health status | 0.809*** | 0.490*** | 0.531*** | 0.595*** | 0.473*** | 0.580*** |
| Controlled variable | | | | | | |
| Current residence (urban area) | − 0.240* | − [illegible]90** | − 0.229* | − 0.369** | − 0.180 | − 0.312** |
| Household type (local rural) | 0.184 | 0.0[illegible] | 0.096 | 0.003 | − 0.332* | 0.159* |
| Gender (male) | 0.001 | 0.[illegible]94 | 0.398** | − 0.201 | − 0.138 | 0.146 |
| Cultural level (primary school and below) | 0.[illegible]55*** | 0.327*** | 0.328*** | 0.356*** | 0.504*** | 0.294*** |
| Age | 0.[illegible]*** | 0.350*** | 0.258* | 0.490*** | 0.359* | 0.403*** |
| Constant | − 6.399[illegible] | − 4.854*** | − 5.309*** | − 5.239*** | − 3.586*** | − 5.665*** |
| -2 log-likelihood | 550.938 | 2470.187 | 1484.270 | 1532.015 | 629.491 | 2384.054 |
| Chi-square | 86.1[illegible]1*** | 234.038*** | 136.023*** | 202.191*** | 68.226*** | 271.734*** |

*$p<0.1$, ** $p<0.05$, *** $p<0.01$, and the number is the coefficient B

RETRACTED ARTICLE

scholars studied the differences in the quality of life of the urban and rural elderly, they found that the differences in the macro policy environment and the social psychology of the elderly are the main reasons for the difference in the quality of life of the urban and rural elderly. The WHO-QOL BREF was used in order to do the evaluation of the quality of life. According to the findings of the research, elderly people living in urban communities reported a significantly lower degree of quality of life in the areas of physical health (51.[illegible]3.6) and psychological well-being (51.3–2.5) than elderly people living in rural areas. Under the guidance of such institutional arrangements and policies, Although rural economic development level, infrastructure construction, medical and health conditions, social security level, public service provision, resident income and other aspects do relatively well, but, There is still a certain gap with the city, therefore, These macro environmental differences with structural and institutional characteristics have had a certain impact on the living environment, living conditions, demand satisfaction and psychological perception of the rural elderly, It then affects life satisfaction and quality of life (Nagata et al., 2021).

## Conclusion

With the deepening of China's aging degree, the country and society pay increasing attention to the elderly group. At present, the material needs of the elderly in China have been basically met, especially in the economically developed areas, the material conditions of the elderly have been met to a greater extent, and the attention to the elderly has gradually changed from the material life to the quality of life. Life-satisfaction of the elderly is the subjective feelings of

RETRACTED ARTICLE

their own life and pension conditions, and also an important measure of the quality of life in their later age (Diener & Diener, 1995). The inherent ability of the elderly is mainly reflected in the physical health and self-care ability, and the value participation is mainly reflected in social labor, community volunteer activities and participation in elderly education. Through the analysis of the inherent ability and value participation of the elderly and life satisfaction of the elderly, the following three conclusions are drawn:

First, one conclusion of this paper is that inherent ability can improve the high quality of life of the elderly. It can be seen from the data analysis that inherent ability has a significant impact on the quality of life and life happiness of the elderly. Inherent ability determines the objective quality of life of the elderly, and plays a decisive role in the happiness and quality of life of the elderly. The government should strengthen the management of sports activities, build sports venues around the community, make the community an important position for developing sports for the elderly; and guide the elderly to form a healthy lifestyle, promote a healthy diet, distribute healthy recipes and cooking teaching for the elderly, etc. In addition to the physical condition of the elderly, the mental health condition of the elderly should also be paid due attention. The establishment of a mental health service system for the elderly in the community can promote the retired elderly to participate in the community mental health services, which also improves the value participation of the elderly.

Second, value participation can enhance the subjective happiness of the elderly, and the subjective initiative of young and middle-aged elderly participation should be promoted to improve the sense of value of the elderly. Participate in intergenerational care can improve the happiness of the elderly is a conclusion of this article, can invite the elderly teaching activities or teaching hosting for children, at the same time, the government promotes the elderly to participate in community governance, in addition to let the elderly participate in community residents' committees management activities, can also promote the elderly to participate in cultural activities, organize painting and calligraphy, tai chi, cooking, vocal music and other cultural activities, to mobilize the enthusiasm of the elderly to participate in community autonomy.

Third, different types of elderly in value participation have different perception, between the elderly gender, health, age, cultural level and living environment, the elderly themselves in different condition, different demand for high quality of life, realize high quality of aging life, need to crowd, according to demand. For example, in the empirical part, the elderly believe that it is happier not to participate in social labor life, so in the process of promoting the value participation of the elderly, the elderly can participate in the family community, such as intergenerational care and neighborhood mediation; while the elderly can focus on social labor, such as echoing the work before retirement.

In the future, based on the above conclusions, we intend to devote ourselves to the path research of high-quality life for the elderly.

**Acknowledgments** The authors extend their appreciation to the Support by the General Project of National Social Science Fund: Research on the Theory, Pathway and Practice Mode of Healthy China Construction (No.: 20BJY014).

**Author Contributions** JL: Editing data curation, Writing—original draft preparation. XC, YD and Y S: Data collection, collation, research and other implementation, put forward my own views, made a great contribution.

**Data Availability** The authors declare that the research was conducted in the absence of any commercial or financial relationships that could be construed as a potential conflict of interest.

## Declarations

**Conflict of interest** The authors declare that the research was conducted in the absence of any commercial or financial relationships that could be construed as a potential conflict of interest.

**Open Access** This article is licensed under a Creative Commons Attribution 4.0 International License, which permits use, sharing, adaptation, distribution and reproduction in any medium or format, as long as you give appropriate credit to the original author(s) and the source, provide a link to the Creative Commons licence, and indicate if changes were made. The images or other third party material in this article are included in the article's Creative Commons licence, unless indicated otherwise in a credit line to the material. If material is not included in the article's Creative Commons licence and your intended use is not permitted by statutory regulation or exceeds the permitted use, you will need to obtain permission directly from the copyright holder. To view a copy of this licence, visit http://creativecommons.org/licenses/by/4.0/.

## References

Borg, C., & Hallberg, I. R. (2006). Life satisfaction among informal caregivers in comparison with non-caregivers. *Scandinavian Journal of Caring Sciences, 20*(4), 427–438.

Brusilovskiy, E., Klein, L. A., & Salzer, M. S. (2016). Using global positioning systems to study health-related mobility and participation. *Social Science & Medicine, 161*, 134–142.

Brusilovskiy, E., Klein, L. A., Townley, G., Snethen, G., McCormick, B., Hiremath, S. V., & Salzer, M. S. (2020). Examining the relationship between community mobility and participation using GPS and self-report data. *Social Science & Medicine*. https://doi.org/10.1016/j.socscimed.2020.113539

Bulanda, J. R., & Jendrek, M. P. (2016). Grandparenting roles and volunteer activity. *Journals of Gerontology Series B: Psychological Sciences and Social Sciences*, *71*(1), 129–140.

Campolina, A. G., et al. (2011). Validação da versão brasileira do questionário genérico de qualidade de vida short-form 6 dimensions (SF-6D Brasil) Validation of the brazilian version of the

RETRACTED ARTICLE

generic six-dimensional short form quality of life questionnaire (SF-6D Brazil). *Ciência & Saúde Coletiva, 16*(7), 3103–3110.

Chen, J., & Wu, Z. (2021). *Gender Differences in the Effects of Self-rated Health Status on Mortality among the Oldest Old in China. Healthy Longevity in China*. Springer.

Chen, Y. Z., & Deng, J. H. (2011). Evaluation of the quality of life of the elderly population in Changchun. *Journal of Population, 06*, 27–34.

Choy-Brown, M., Stanhope, V., Williams, N., & Bond, L. (2020). Delivering person-centered care in community mental health programs. *Research on Social Work Practices, 30*(8), 907–917.

Chunyu, Li., Linxi, T., Jinzhen, J., & Li, Z. (2021). The influence of intrinsic abilities and external environmental factors on healthy aging in community-dwelling older adults[J]. *Journal of Liberation Army Nursing, 38*(12), 22–25.

Coke, M. M. (1992). Correlates of life satisfaction among elderly African Americans. *The Journal of Gerontology, 1992*, 47.

de Souza Macêdo, R. P., et al. (2021). Possible association of early menopause with worse physical function: a systematic review. *Menopause*. https://doi.org/10.1097/GME.0000000000001712

Diener, E., & Diener, M. (1995). Cross cultural correlates of life satisfaction and self-esteem. *Journal of Personality and Social Psychology, 68*(4), 653–663.

Dongfang, M. (2021). Residents' perceptions and creation paths of high quality of life-an analysis based on a survey of 39 districts and counties in Chongqing. *Western Forum, 31*(03), 44–56.

Dury S, De Donder L, De Witte N, et al. (2014a).To Volunteer or Not: The Influence of Individual Characteristics, Resources, and Social Factors on the Likelihood of Volunteering by Older Adults. Science Letter, 2013

Eunjin, L., & Junpyo, K. (2021). Effects of oral health on life satisfaction in older adults: Moderated mediating effects of subjective health and social activities. *Korean Journal of Convergence Science*, *10*(4), 224–241.

Glass, T. A., De Leon, C. F., Bassuk, S. S., & Berkman, L. [illegible] (2015). Social engagement and depressive symptoms in Late Life [illegible]gitudinal findings. *Journal of Aging and Hea*[illegible], 604–62[illegible].

Guangzong, M. (2019). Quality pension: the new pursu[illegible] of the new generation of urban elderly. *People's Forum, 13*, 72–[illegible].

Helliwell, J. F., & Putnam, R. D. (2004). The social context of well-being. *Philosophical Transactions of the Royal Society, 359*, 1435–1446.

Huang, F., & Fu, W. (2017). Government purchase or home care? – An empirical analysis based on [illegible]tution effect of family caregiving. *Nankai Economic Research, 01*, 136–152.

Jiaming, Z., Qiuyun, L., [illegible]ng, Z., Yong, W., Zhengdong, W., Xuefeng, C., Degu[illegible], [illegible]g, W., & Li, J. (2016). The mediating effect of emo[illegible]nal balance between health status and life satisfaction in long-li[illegible] elderly: An application of structural equation [illegible]eling. *Journal of Fudan (medical Edition), 43*(01), 16–21.

Jiang, M. M., Tan, L., & [illegible]ang, Y. M. (2018). The effects of self-care abil[illegible] [illegible]ging attitudes on the well-being of older adults. *Chinese Jou[illegible] of Gerontology, 38*(05), 1230–1233.

Kim, [illegible]. (202[illegible]). Effect of the awareness of calling, wisdom, and job sati[illegible] on the quality of services of care workers in charge of [illegible]iting care under the care center of the elderly. *Proceedings of the* [illegible]*ciety for Sexual and Human Health*, 115.

Lin, M. H., Chen, L. J., Huang, S. T., Meng, L. C., Lee, W. J., Peng, L. N., Hsiao, F. Y., & Chen, L. K. (2022). Age and sex differences in associations between selfreported health, physical function, mental function and mortality. *Archives of Gerontology and Geriatrics*, *98*, 104537.

Linfeng, L., Lingna, Y., Hongyan, W., Qin, L., Qin, L., & Xiangeng, Z. (2020). Correlation between mental health and subjective well-being of elderly people in nursing institutions. *Chinese Journal of Gerontology, 40*(24), 5277–5280.

Nagata, S., McCormick, B., Zisman-Ilani, Y., Wilson, S., Snethen, G., Townley, G., & Salzer, M. S. (2021). Emotional states associated with being in the community and being with others among individuals with serious mental illnesses. *American Journal of Orthopsychiatry, 91*(1), 1–8.

Noel, V. A., Oulvey, E., Drake, R. E., & Bond, G. R. (2017). Barriers to employment for transition-age youth with developmental and psychiatric disabilities. *Administration* [illegible] *Policy in Mental Health and Mental Health Services Research, 4*[illegible], 35[illegible]–358.

Noguchi, T., Ishihara, M., Murata, C., Nakagawa, T., [illegible]omatsu, A., Kondo, K., & Saito, T. (2022). Art [illegible] cultural activity engagement and depressive symptom ons[illegible] amo[illegible] older adults: A longitudinal study from the Japanese Gerontological Evaluation Study. *International Journal of Geriatric Psychiatry*, *37*(3). https://doi.org/10.1002/gps.5685

Palmes, M. S., Trajera, S. M., [illegible] Ching, G. S. (2021). Relationship of coping strategies and quality of life: Parallel and serial mediating role of resilience and social participation among older adults in Western Philippines. *International Journal of Environmental Research and Public Health*, *18*(19), 10006. https://doi.org/10.3390/ijerph18[illegible]10006

Park, M. H., [illegible] Sok, S. (2022). Perception, knowledge and attitudes on advance medical directives among hospital staff: Using mixed methodology. *Journal of Clinical Nursing*, *31*(17-18), [illegible]631.

Resolution of the CPC Central Committee on the Major Achievements and Historical Experience of the Party's Hundred Years, November 2021.

[illegible]n, J. (2021). Measuring the health of the elderly as a function. *Jinqiu, 10*, 50.

Shiba, K., Torres, J. M., Daoud, A., Inoue, K., & Kawachi, I. (2021). Estimating the impact of sustained social participation on depressive symptoms in older adults. *Epidemiology*, *32*(6), 886–895.

Skla, B., Xdl, C., Me, D., et al. (2021). A longitudinal study on social support, social participation, and older Europeans' quality of life. *SSM-Population Health, 13*, 2021.

Song, J., Wang, J. W., & Qin, T. T. (2018). A study on the relationship between grandchild care and employment of older adults. *Population and Economy, 03*, 92–103.

The Global Report on Aging and Health,, World Health Organization, 2016.

The Law of the People's Republic of China on the Protection of the Rights and Interests of the Elderly was amended on December 29,2018.

Townley, G., Brusilovskiy, E., & Salzer, M. S. (2017). Urban and non-urban differences in community living and participation among individuals with serious mental illnesses. *Social Science & Medicine, 177*, 223–230.

Townley, G., Brusilovskiy, E., Snethen, G., & Salzer, M. S. (2018). Using geospatial research methods to examine resource accessibility and availability as it relates to community participation of individuals with serious mental illnesses. *American Journal of Community Psychology, 61*(1–2), 47–61.

Townley, G., Terry, R., Snethen, G., & Salzer, M. S. (2019). Associations between physical health limitations and community participation among people with and without serious mental illnesses. *Psychiatric Services, 70*(8), 689–695.

Wang, S. B., Shen, Q., & Wang, M. Z. (2015). An empirical study of social participation of mobile elderly in comparative perspective. *Southern Population, 30*(05), 44–51+80.

Wong, F. M. F. (2019). Life satisfaction and quality of life enjoyment among retired people aged 65 or older. *Open Journal of Social Sciences, 7*(5), 119–127.

World Health Organization.World Report on Ageing and Health[R]. Geneva: World Health Organization,2016.

Wu, W. G., & Huang, L. (2021). A discussion on value engagement in high school counseling and guidance. *China Teacher, 07*, 106–107.

Xiaolan, Wu., & Ji, L. (2018). A study on the developmental trajectory of elderly people's self-care ability in China. *Journal of Population, 40*(04), 59–71.

Xie, L. L., & Wang, B. (2019). Social participation patterns and influencing factors of Chinese older adults in the perspective of active aging. *Population Research, 43*(03), 17–30.

Yafang, L. (2011). An empirical analysis of subjective well-being of the elderly. *Journal of Nanjing Population Management Cadre College, 27*(02), 35–40.

Yan, W., He, M. J., Lu, Y., & Chang, F. (2021). A study on the current status of elderly disability and its influencing factors based on CLHLS in China. *Nursing Research, 35*(10), 1807–1811.

Yue, Z., Qiaodan, X., Xi, L., Yao, L. T., Ying, B. J., Nan, L. R., Ying, W., & Hong, S. (2021). A study on life satisfaction and its influencing factors among Chinese elderly people. *China Rural Health Management, 41*(10), 729–733.

Zhang, W. J., & Zhao, D. Y. (2015). A study on the social participation patterns of low-aged elderly in urban areas[J]. *Population and Development, 21*(01), 78–88.

Zimmermann, K. F. (1991). Ageing and the labor market: age structure, cohort size and unemployment. *Journal of Population Economics, 4*(3), 177–200.


**Publisher's Note** Springer Nature remains neutral with regard to jurisdictional claims in published maps and institutional affiliations.

RETRACTED ARTICLE
